# Resolution of Pulmonary Inflammation and Obstruction Following Repair of Cerebrospinal Fluid Leak: A Case Report and Review of the Literature

**DOI:** 10.1155/crpu/9651572

**Published:** 2026-01-07

**Authors:** Ann O. Birmingham, Serban A. Staicu, Isaac L. Schmale, Li-Xing Man

**Affiliations:** ^1^ University of Rochester School of Medicine & Dentistry, Rochester, New York, USA, rochester.edu; ^2^ Division of Pulmonology and Critical Care Medicine, University of Rochester Medical Center, Rochester, New York, USA, rochester.edu; ^3^ Department of Otolaryngology Head and Neck Surgery, University of Rochester Medical Center, Rochester, New York, USA, rochester.edu

**Keywords:** aspiration, cerebrospinal fluid rhinorrhea, CSF rhinorrhea, pneumonitis, runny nose

## Abstract

**Objectives:**

Cerebrospinal fluid (CSF) rhinorrhea involves drainage of CSF through the nasal cavity due to disruption of the skull base, and pneumonitis secondary to CSF aspiration is a lesser‐known complication reported. We present the case of a patient whose pulmonary symptoms and function improved after surgical CSF leak repair.

**Methods:**

This is a case report with review of the electronic medical record and literature review.

**Results:**

A 35‐year‐old female presented with 1 year of refractory right‐sided rhinorrhea and recurrent lower respiratory infections. Nasal drainage was positive for beta‐2‐transferrin. Sinus CT showed a possible leakage site at the right lateral lamella of the cribriform plate. Chest CT showed upper lobe predominant ground‐glass opacities. Following surgical CSF leak repair, chest CT showed resolution of ground‐glass opacities and pulmonary function testing showed an increase in spirometric lung volumes.

**Conclusions:**

Aspiration pneumonitis from CSF leaks is rarely reported. Studies report resolution of ground‐glass opacities on chest CT as well as clinical improvement in symptoms such as cough following surgical leak repair. Proposed mechanisms leading to aspiration pneumonitis include nasopharyngeal bacterial transit; the mechanism of persistent leak may be related to cough‐induced increase in intracranial pressure leading to increased CSF flow. In patients with lower airway inflammation and obstruction as well as unexplained aspiration pneumonitis, a high index of suspicion for CSF rhinorrhea is warranted.

## 1. Introduction

Cerebrospinal fluid (CSF) rhinorrhea involves drainage of CSF through the nasal cavity due to disruption of the skull base [1]. Most cases are secondary to trauma, either accidental or iatrogenic, but may also develop spontaneously [1, 2]. In addition to rhinorrhea, patients may also present with headache, symptoms of rhinosinusitis or allergic rhinitis, tinnitus, and visual or olfactory disturbances [1, 3]. A serious complication of CSF rhinorrhea includes ascending infection leading to meningitis; thus, surgical repair is usually recommended [4, 5]. To confirm the diagnosis, beta‐2‐transferrin testing of nasal discharge is performed, which is known to have high specificity and sensitivity [4]. Following a positive beta‐2‐transferrin test, imaging is performed to evaluate for structural abnormalities and localize the source [2, 3].

Aspiration pneumonitis involves inflammation of lung tissue, usually due to the entry of gastric or oropharyngeal contents into the lower respiratory tract [6, 7]. In addition, there is evidence that sinonasal secretions may be aspirated into the lungs and cause irritation [3, 8]. Thus, a lesser‐known complication of CSF rhinorrhea is aspiration pneumonitis secondary to CSF aspiration [1, 3, 7, 9–11]. We report the case of a patient with improvement in pulmonary symptoms and pulmonary function testing following endoscopic transnasal CSF leak repair.

## 2. Case Description

A 35‐year‐old female presented with approximately 1 year of clear, right‐sided rhinorrhea complicated by recurrent lower respiratory infections requiring oral antibiotics and steroid treatment. Of note, she sustained trauma to the back of her head during a syncopal episode approximately 1 month prior to the onset of her rhinorrhea and first episode of pneumonia. Her medical history included allergic rhinitis, obstructive sleep apnea, gastroesophageal reflux disease, and obesity (body mass index 53.1).

She was initially referred to allergy/immunology for right‐sided rhinorrhea unresponsive to oral cetirizine and fluticasone and azelastine sprays. Her blood allergy testing was positive for cockatiel feathers only. For her recurrent flares of cough and dyspnea, the patient was evaluated by pulmonology. Pulmonary function tests (PFTs) revealed reduced FEV_1_ and FVC with preserved FEV_1_/FVC ratio (Table 1). Inhaled corticosteroid and bronchodilator therapy was initiated for a tentative diagnosis of asthma associated with environmental allergies. Montelukast was also added to her regimen. The patient was then evaluated by otolaryngology for her persistent, nonallergic right‐sided rhinorrhea. She described the drainage as salty, thin, and pouring out of her nose in certain positions. Nasal drainage was collected for beta‐2‐transferrin testing, which was positive.

**Table 1:** Pulmonary function testing pre‐ and post‐repair of CSF leak.

**Date**	**FEV**_**1**_	**FVC**	**FEV**_**1**_ **/FVC**
10/7/2022 (pre‐repair)	2.3 L	2.7 L	85%
11/30/2023 (post‐repair)	2.74 L 19% increase	3.29 L 22% increase	83%

**Figure 1:**
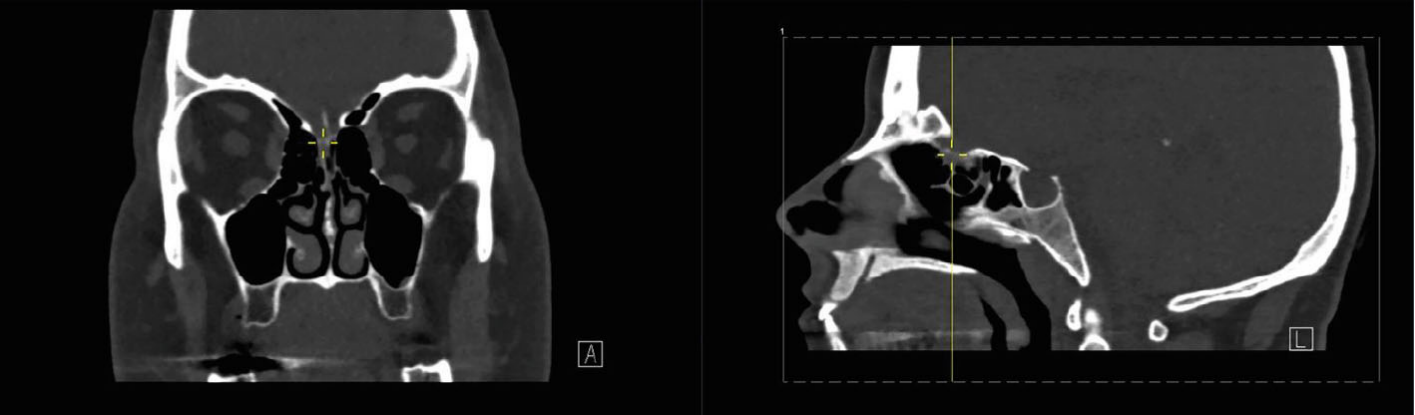
Preoperative coronal and sagittal CT images of a possible CSF leakage site at the right lateral lamella of the cribriform plate with no obvious (meningo)encephalocele.

Sinus CT showed no obvious encephalocele or meningocele and no skull base defect in the sphenoid but revealed a possible leakage site at the right lateral lamella of the cribriform plate (Figure 1). An empty sella was also noted, raising suspicion for idiopathic intracranial hypertension (IIH). However, upon ophthalmology evaluation, no papilledema was noted, and thus, her CSF leak was not suspected to be secondary to IIH. Chest imaging obtained at that time revealed upper lobe predominant patchy parenchymal ground‐glass opacities (Figure 2) as well as lingular consolidation.

Figure 2Preoperative chest CT with upper lobe predominant patchy parenchymal ground‐glass opacities (a). Postoperative chest CT taken 4 months after surgery with interval resolution in ground‐glass opacities (b).

**Figure:**
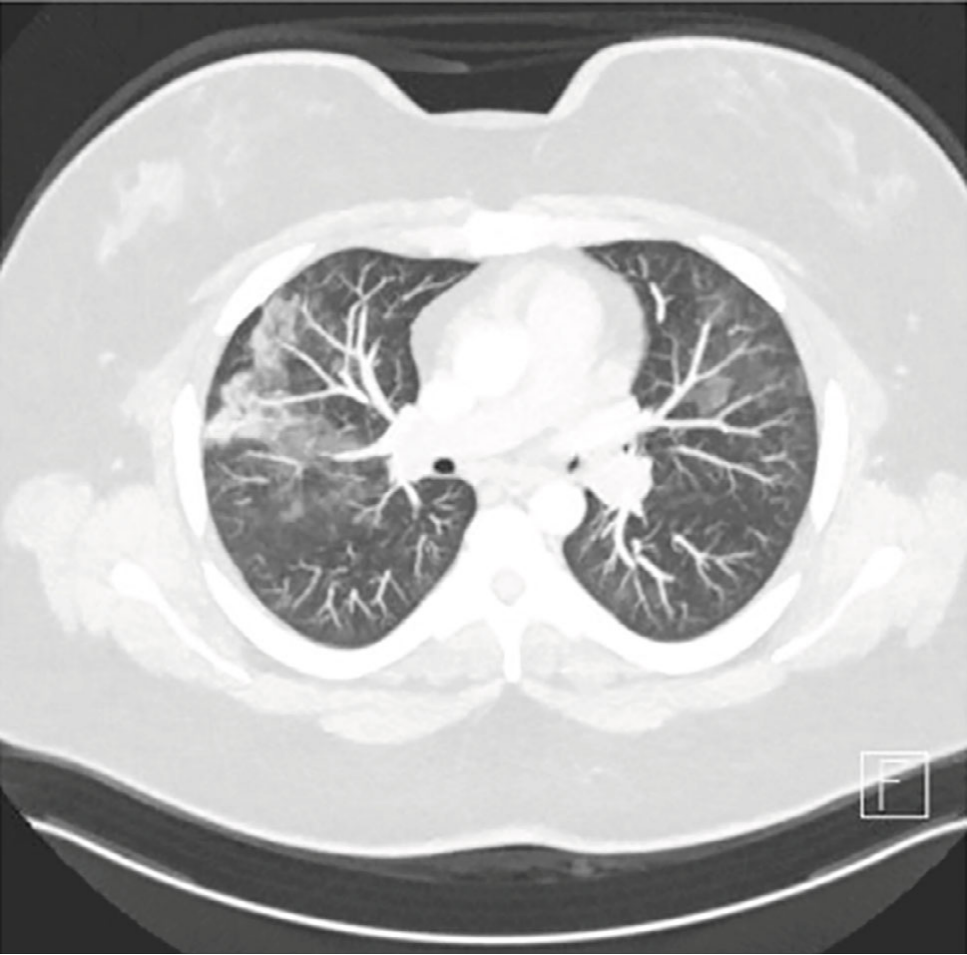
(a)

**Figure:**
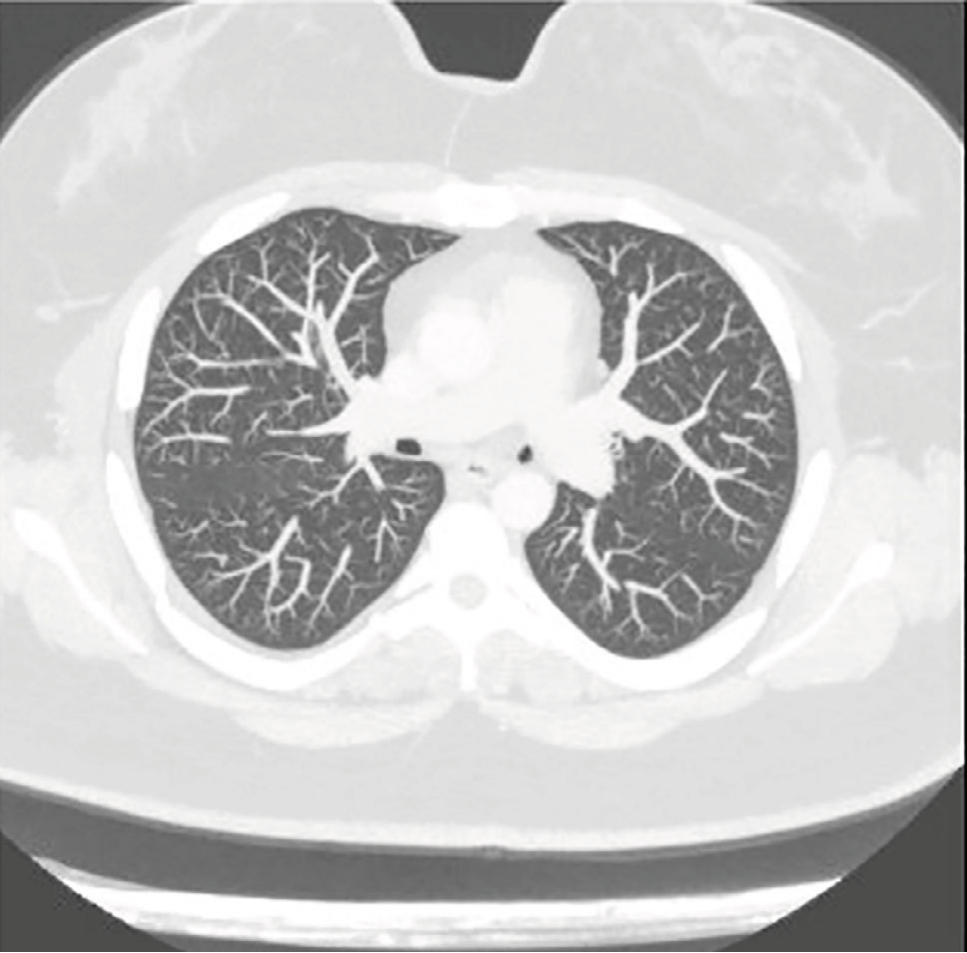
(b)

The patient then underwent right transnasal CSF leak repair at the posterior lateral lamella of the cribriform plate with a left nasal floor free mucosal graft. Operative findings included a small area of encephalocele versus edematous mucosa at the CSF leak site, as well as a 1 × 2 mm skull base defect of the right cribriform plate just medial to the attachment of the superior turbinate. No evidence of CSF rhinorrhea was noted during her hospital stay. She did well postoperatively and denied recurrence of nasal drainage suggestive of CSF releakage at subsequent otolaryngology follow‐ups. She did report periorbital headaches, for which she took topiramate and was referred to neurology.

Repeat chest CT 4 months after surgery showed interval resolution of patchy ground‐glass opacities and lingular consolidation (Figure 2). Following her surgery, the patient self‐discontinued her inhaled therapy but continued montelukast and antihistamines for her history of allergic rhinitis. The patient continued to do well, reporting no dyspnea, wheezing, or chest tightness at pulmonary follow‐up 8 months after surgery. A substantial increase in spirometric lung volumes was noted on pulmonary function testing compared to presurgery values, including a 19% increase in FEV_1_ and a 22% increase in FVC (Table 1).

The patient′s pulmonary symptoms were initially thought to be asthma induced, but her symptoms did not resolve until her CSF leak repair was performed. Thus, in retrospect, her symptoms were felt to be related to chronic CSF aspiration leading to pneumonitis, further supported by substantial improvement in PFTs and resolution of ground‐glass opacities on chest imaging on repeat testing after her CSF leak repair.

## 3. Discussion

A recent study reported the first estimate of the prevalence of aspiration pneumonitis resulting from spontaneous CSF rhinorrhea as 25.2% [7]. However, because it is not routine to undergo chest CT following the diagnosis of CSF leak, the true prevalence of aspiration pneumonitis in patients with CSF leaks is likely underdiagnosed, with pulmonary symptoms underrecognized in comparison to neurological complications [7]. Consequently, delays exist in diagnosis and treatment [1, 10]. Indeed, in our presented case, the time between referral to allergy/immunology and beta‐2‐transferrin testing positivity was 219 days.

As stated above, chronic aspiration pneumonitis secondary to CSF rhinorrhea has been reported in the literature [1, 3, 7, 9–11]. Seltzer et al. report the case of a patient with confirmed CSF leak with resolution of cough, exertional dyspnea, and hoarseness following endoscopic repair [10]. The case series by Or et al. found that symptomatic chronic pneumonitis was present in 6 of 20 patients with spontaneous CSF rhinorrhea, with ground‐glass opacities identified on chest CT for all 6 patients. Surgical repair resolved both symptoms of pneumonitis and ground‐glass opacities on chest imaging in all cases. Thus, the argument is made that CSF rhinorrhea should be considered in cases of nonresolving chronic pneumonitis, especially if clinical signs and symptoms support the diagnosis [3]. Similarly, Cao et al. report complete resolution of pneumonitis following surgical repair of spontaneous sphenoid sinus meningoencephalocele with CSF rhinorrhea [9]. Lastly, Jones et al. report progressive clinical improvement of cough and pleuritic chest pain following endoscopic repair in a patient with CSF rhinorrhea and a history of head trauma. Notably, during induction of anesthesia, aspiration of a large volume of clear nasopharyngeal fluid was observed [11].

Chronic aspiration pneumonitis secondary to CSF rhinorrhea may also be asymptomatic. Takekoshi et al. report two cases of nonresolving pneumonia where ground‐glass opacities on chest CT resolved completely following resolution of CSF rhinorrhea via surgical repair. Unlike most reported cases, one patient experienced persistent rhinorrhea only, without lower respiratory symptoms despite the presence of lung infiltrates on chest imaging [1].

There are several proposed mechanisms through which CSF rhinorrhea leads to aspiration pneumonitis and pulmonary symptoms. Aspiration of CSF may lead to inflammation or pneumonia due to the transit of nasopharyngeal bacteria [11]. Determining whether the characteristics of CSF itself lead to pulmonary mucosal damage or inflammation requires further investigation. Furthermore, cough, as experienced by most reported patients with aspiration pneumonitis, increases intracranial pressure and may increase the flow of CSF, thus leading to continued aspiration and lung inflammation [9].

In addition to the above cases, our case implicates CSF in pneumonitis, as evidenced by the resolution of ground‐glass opacities on imaging. Furthermore, aspirated CSF rhinorrhea may also directly contribute to lower airway inflammation and obstruction, demonstrated by the patient′s improvement on spirometry postrepair and self‐discontinuation of inhaled corticosteroid and bronchodilator therapy. To the authors′ knowledge, only one existing case describes the resolution of abnormal PFTs following endoscopic CSF leak repair [11]. In our presented case, endoscopic CSF leak repair resulted in measurable increases in both FEV_1_ and FVC, indicating improvement in lower airway irritation. Thus, aspirated CSF contributes to not only inflammation of the lung parenchyma but also airflow obstruction that can be measured on spirometry.

## 4. Conclusion

Reports of aspiration pneumonitis associated with CSF leaks are rare, with only one reported case describing abnormal PFTs. In most reported cases, surgical repair led to clinical improvement with resolution of abnormal pulmonary imaging findings. In patients with lower airway inflammation and obstruction as well as findings of unexplained aspiration pneumonitis, a high index of suspicion for CSF rhinorrhea is warranted.

## Ethics Statement

Our institution does not require ethical approval for reporting individual cases. No patient protected health information is included in this article.

## Conflicts of Interest

The authors declare no conflicts of interest.

## Funding

No funding was received for this manuscript.

## Data Availability

The authors have nothing to report.
